# A screen of small molecule and genetic modulators of life span in female *Drosophila* identifies etomoxir, RH5849 and unanticipated temperature effects

**DOI:** 10.1080/19336934.2022.2149209

**Published:** 2022-11-22

**Authors:** Gary N. Landis, Sebastian Ko, Oscar Peng, Brett Bognar, Michael Khmelkov, Hans S. Bell, John Tower

**Affiliations:** Molecular and Computational Biology Section, Department of Biological Sciences, University of Southern California, Los Angeles, CA, USA

**Keywords:** Ageing, pioglitazone, steroid, mifepristone, GAL4/GAL80ts, ecdysone, climate change, sex differences

## Abstract

Mifepristone increases life span in female *Drosophila melanogaster*, and its molecular target(s) remain unclear. Here small molecule and genetic interventions were tested for ability to mimic mifepristone, or to decrease life span in a way that can be rescued by mifepristone. Etomoxir inhibits lipid metabolism, and significantly increased life span in virgin and mated females, but not males, at 50 µM concentration. Pioglitazone is reported to activate both mammalian PPARγ and its *Drosophila* homolog Eip75B. Pioglitazone produced minor and inconsistent benefits for female *Drosophila* life span, and only at the lowest concentrations tested. Ecdysone is a *Drosophila* steroid hormone reported to regulate responses to mating, and RH5849 is a potent mimic of ecdysone. RH5849 reduced virgin female life span, and this was partly rescued by mifepristone. Mifepristone did not compete with RH5849 for activation of an ecdysone receptor (EcR)-responsive transgenic reporter, indicating that the relevant target for mifepristone is not EcR. The conditional GAL4/GAL80ts system was used in attempt to test the effect of an *Eip75B* RNAi construct on female life span. However, the 29°C temperature used for induction reduced or eliminated mating-induced midgut hypertrophy, the negative life span effects of mating, and the positive life span effects of mifepristone. Even when applied after mating was complete, a shift to 29°C temperature reduced mating-induced midgut hypertrophy by half, and the life span effects of mating by 4.8-fold. Taken together, these results identify promising small molecules for further analysis, and inform the design of experiments involving the GAL4/GAL80ts system.

## Introduction

Mifepristone is a synthetic steroid, with a long history of safe use in humans [1,2]. Mifepristone is used for birth control, based on its ability to antagonize the progesterone receptor. Mifepristone is also used to treat Cushing’s disease (hypercortisolism), based on its ability to antagonize the type II glucocorticoid receptor (GR). More recently, mifepristone has become of additional interest due to its anti-cancer, anti-obesity, and anti-diabetes effects in human and rodent models [3–5]. For example, short-term inhibition of the GR with mifepristone was reported to improve insulin sensitivity in overweight and pre-diabetic subjects [5]. Mifepristone is also a mammalian PPARγ agonist that activates expression of PPARγ target genes [6,7]. Mifepristone improved insulin sensitivity and adiponectin levels in mice fed a high-fat diet, and caused adiponectin release from cultured adipocytes that was dependent upon PPARγ [8]. The identification of mammalian PPARγ as a mifepristone target is of particular interest. PPARγ is a central regulator of lipid and glucose metabolism [9], and acts as an anti-inflammatory regulator in the gut [10,11], and as a regulator of lipid metabolism adaptation during pregnancy [12]. Thiazolidinedione drugs, including pioglitazone and rosiglitazone, have been used to treat diabetes based on their ability activate PPARγ [9,13]. *Drosophila* has a PPARγ homolog called Eip75B (75B), which is required for mating-induced midgut remodelling [14,15]. Both mammalian PPARγ and *Drosophila* 75B are reported to be activated by pioglitazone, and have similar ligand-binding domains [14,16].

*Drosophila* has proven to be a promising model for the study of mifepristone and its effects on metabolism and midgut plasticity. In female *Drosophila*, mating and male Sex Peptide (SP) hormone cause increased levels of the sesquiterpenoid hormone juvenile hormone (JH), and the steroid hormone 20-hydroxyecdysone (ecdysone) [17]. JH and ecdysone induce intestinal stem cell (ISC) proliferation, midgut hypertrophy, and increased AA and lipid metabolism [14,15,18], which supports increased egg production [19,20]. The mechanism for ecdysone involves activation of the ecdysone receptor (EcR), and EcR in turns acts as a transcriptional activator to increase expression of 75B in the midgut [14,15]. Increased expression and activity of 75B promotes ISC proliferation and daughter cell differentiation, thereby contributing to midgut hypertrophy. Ageing in female and male *Drosophila* is associated with further ISC proliferation and mis-differentiation (often called ‘dysplasia’), regulated in part by oxidative stress, EGFR signalling, and the microbiome [21–23].

The midgut hypertrophy, increased AA metabolites and lipids, inflammation, and decreased life span caused by mating and SP can each be reversed by feeding the mated females mifepristone, yielding +100% increase in median life span [24–26]. It is therefore of interest to determine what are the specific mifepristone targets and mechanisms that enable mifepristone to block the effects of mating. Moreover, mifepristone also increases life span of virgin females by +16-30% [24,27], but does so without detectable alteration in midgut size [27]. It is therefore also of interest to determine the mifepristone targets and mechanisms in virgin females, and how these might be related to the mifepristone targets and mechanisms in mated females. Effects of mifepristone in *Drosophila* do not require presence of Gene-Switch transgenes [24,25], and no antibiotic activity was detected for mifepristone [28,29]. Remarkably, mifepristone often uncouples *Drosophila* life span from food intake. Food intake has been measured using the dye-uptake assay [25], the CAFÉ assay [24], and the EXQ assay [26,27], and in each case mifepristone caused either no change or a significant increase in food consumption. For example, mifepristone increased life span of virgin females fed the JH analog methoprene by +80%, while simultaneously doubling food intake [26]. Similarly, mifepristone increased life span of virgin females on both normal and high-fat diets, while simultaneously increasing food intake [27]. Several life span-extending interventions in *Drosophila* have greater effect in short-lived starting strains, for example, adult-specific over-expression of Cu/ZnSOD [30]. In contrast, mifepristone life span extension is unusual in that it is greatest in long-lived starting strains [25]. To further investigate possible mifepristone mechanisms, several small molecules and a genetic intervention were screened for the ability to mimic mifepristone, or to decrease life span in a way that can be rescued by mifepristone.

## Materials and methods

### *Drosophila* strains and culture

*Drosophila melanogaster* were cultured using a standard agar/dextrose/corn meal/yeast media [31], at 25°C (unless indicated otherwise), and adult flies were passaged to fresh media every-other day. Several strains were obtained from Bloomington *Drosophila* Stock Center. w[*]; EcRE-lacZ[SS3] (BDSC#4517). Strain y[1] w[*]; P{w[+mC] = elav-Switch.O}GSG301 (BDSC#43642), which is abbreviated here as *y; Elav-GS*. The *w[1118]* strain is the isogenized version (*w[1118]- iso; 2-iso; 3-iso*) which was previously cured of Wolbachia by three generations treatment with doxycycline, with confirmation using PCR and Wolbachia-specific primers [32]. Two driver strains were used for the GAL4/GAL80ts conditional system [33]. The *αTubulin* gene based driver was *w[1118];P{w[+mC] = tubP-GAL80[ts]}10; P{w[+mC] = tubP-GAL4}LL7/TM6B, Tb[1]*, (BDSC#86328), here abbreviated as ‘tub’ driver. The *escargo* gene based driver was the esgReDDM strain, genotype *w[1118]; esg-Gal4, UAS-mCD8::GFP/CyO; tub-Gal80ts, UAS-H2B::RFP/TM3 Ser*, generously provided by Tobias Reiff [20,34], and here abbreviated as ‘esg’ driver. The strain *w[1118]; UAS-Eip75B-RNAi* was obtained from Vienna *Drosophila* Resource Center (VDRC#44851).

### *Drosophila* life span assay and food-intake assay

To generate flies for life span assays, unless indicated otherwise, *w[1118]* strain males were crossed to *y; Elav-GS* strain virgin females, cultured at 25°C, and hybrid female progeny were collected as virgins over 24 hours. These hybrid progeny are used because of their long starting life span, and their robust response to mating effects on life span [24,25]. For GAL4/GAL80ts system experiments, ‘tub’ or ‘esg’ drivers were crossed to either *w[1118]* or to *UAS-Eip78-RNAi* strain, as indicated, cultured at 18°C, and progeny lacking the balancer chromosome were selected. For the GAL4/GAL80ts system experiments, the newly-eclosed flies were shifted to 29°C within 24 hours. For the other temperature shift experiments, the flies were shifted to 29°C either before or after the 48 hour mating period, as indicated. Flies were either assayed as virgins, or were mated for 48 hours to young (1–2 wk of age) *w[1118]* males at a ratio of 20 males to 20 females. For the GAL4/GAL80ts system experiment with the ‘tub’ driver, the *w[1118]* males used for mating were shifted to 29°C coincident with the start of mating, whereas for the ‘esg’ driver experiment, the *w[1118]* males were shifted to 29°C 48 hours prior to the beginning of mating. After mating, the males were removed, and flies were maintained in culture vials in the presence/absence of drug, as indicated. Drugs were administered as previously described, by applying 100 μl of 10X stock solution in water, or 50 μl of 20X stock solution in ethanol, evenly to the surface of the vial, and allowing to absorb and dry overnight [31,35]. Final concentration of drug in the media was calculated based on absorption into the top ∼1 ml of media, as determined by dye-absorption controls [31,35]; control vials received equal volume of water or ethanol vehicle, and all vials were allowed to dry overnight. Mifepristone (RU486) was obtained from Sigma-Aldrich (cat. #M8046), and flies were treated with 200 μg/ml final concentration in the media. RH5849 (1,2-Dibenzoyl-1-(t-butyl)hydrazine) was obtained from AABLOCKS (cat. #AA00832D). Etomoxir was obtained from Cayman Chemical (cat. #11969). Pioglitazone was obtained from VWR/Avantor (cat. #BT135515). Median life span, percent change in median, log-rank *p* values were conducted using R statistical environment [36]. Food intake was measured using the excreta quantification (EXQ) assay. EXQ assay was conducted essentially as initially described [37], with minor modifications as previously described [26]. Briefly, assays were conducted using 10 flies per assay chamber, and four replicate assay chambers per sample, at d 12 of drug treatment. Dye concentration in dissolved excreta was quantified relative to a standard curve using the spectrophotometer, and data is plotted as mean ± Standard Deviation in bar graphs. ANOVA analyses were conducted using Prism 9, and multiple comparisons were controlled using Tukey’s correction. For all other experiments, multiple comparisons were controlled using Bonferroni correction. The *p* value for significance at 5% error rate is indicated in the figure legends.

### *Drosophila* midgut diameter assay

The maximum midgut diameter was assayed as previously described [27]. Briefly, midguts were dissected in PBS in groups of five flies at a time, and immediately mounted on slides with coverslip spaced using double-stick tape [38]. Visible light images were generated using Leica MZFLIII microscope and SPOT imaging software, and analysed using ImageJ. The maximum diameter region(s) of each midgut sample were estimated by inspection, multiple measurements in those region(s) were generated using ImageJ, and the largest value was used for analysis. ANOVA was conducted using Prism 9, and multiple comparisons were controlled using Tukey’s correction. The *p* value for significance at 5% error rate is indicated in the figure legends.

### Ecdysone receptor (EcR)-responsive lacZ reporter assays

Virgin females of strain w[*]; EcRE-lacZ[SS3] were collected over 24 hours and then maintained at 15 per vial, in the presence or absence of the indicated drugs. On d 6 the flies were transferred to empty vials containing a Kimwipe wetted with deionized water overnight, to clear the flies guts of media. Three replicate extracts of three flies each were then generated for each group. Beta-galactosidase activity was assayed as previously described [39,40], using chromogenic substrate CPRG (Sigma-Aldrich Cat#10,884,308,001), at 37°C for 1 hour. Product was quantified at 595 nm using the Nanodrop, under conditions where activity was linear with regard to time and amount of extract. Protein concentration of the extracts was measured using Bradford reagent (Bio-Rad, Cat#5,000,205), and beta-galactosidase activity was normalized to extract protein. Data are presented in bar graphs as mean ±SD of the three replicate extracts for each group.

## Results and discussion

### Etomoxir

**Figure 1.**
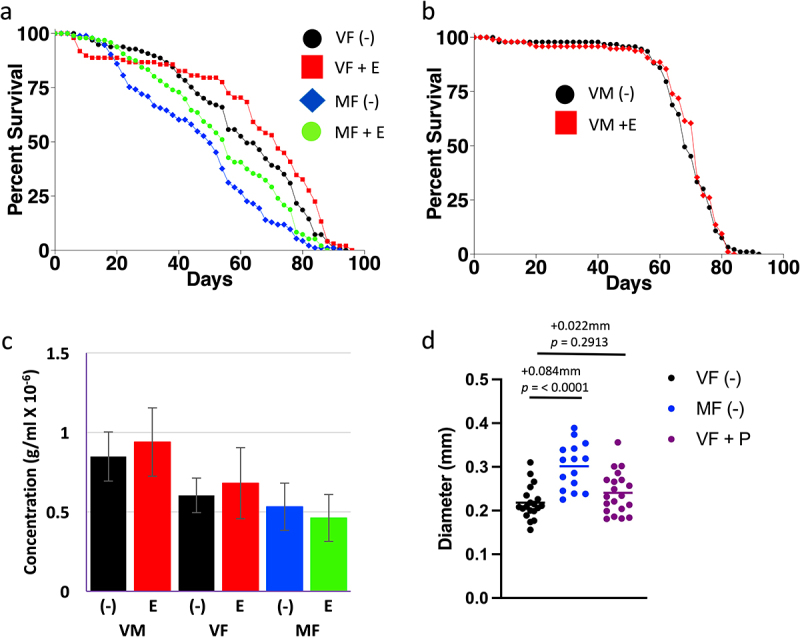
Assays of etomoxir and pioglitazone. (a-c) Effect of 50 µM etomoxir on life span and food intake. (a) Female life span. (b) Male life span. Statistical summary for life span presented in Table 1, Experiment replicate 1. (c) Female and male EXQ food intake assay. Bars indicate average and standard deviation of 4 replicates. Statistical assay is unpaired, two-sided t test, and the *p* value for significance with one comparison is *p* = 0.05. No significant differences were observed between minus-drug and etomoxir-treated groups. (d) Effect of mating and pioglitazone on virgin female midgut diameter. Statistical summary for ANOVA and pair-wise comparisons presented in Table S1; the corrected *p* value for significance is *p* = 0.05. The change in mean maximum midgut diameter between samples and the *p* value for significance is presented above the plots. VF, virgin female. MF, mated female. VM, virgin male. (-), no drug. E, 50 µM etomoxir. P, 200 µg/ml pioglitazone.

Previous results suggest the hypothesis that mifepristone may increase life span in part through inhibition of lipid metabolism. Metabolomics assays showed that mating increased whole-body levels of several lipids, and this was inhibited by mifepristone [26]. In addition, mifepristone partially rescued the negative life span effects of a high-fat diet (HFD) in both virgin and mated females [27]. Finally, mifepristone was reported to reduce lipid uptake in flies of undefined sex [41]. Etomoxir is an irreversible inhibitor of carnitine palmitoyltransferase I (CPT I), the rate-limiting enzyme for transport of long-chain FAs into the mitochondria [42]. Etomoxir (25–100 µM) has been used in *Drosophila* to inhibit FA oxidation during sleep studies [43,44], and is reported to inhibit growth of ISC tumours [45]. Here, etomoxir was tested for life span effects in virgin females, mated females and virgin males, at 50 µM concentration in two replicate experiments (Figure 1a,b; Figure S1a,b; Table 1). Etomoxir significantly increased life span in virgin females (+3.4% and +16.1%, respectively), and in mated females (+22.9% and +12%, respectively; Figure 1a; Figure S1a; Table 1). Etomoxir had no effect on life span in males in either replicate (Figure 1b; Figure S1b; Table 1), similar to the female-specific effects of mifepristone. At 100 µM concentration, etomoxir had no significant effect on mated females, and decreased life span in virgin females in one of the two replicates (−8.33%; Figure S1c,d; Table 1). These data suggest that etomoxir can increase female life span at moderate concentration, but is neutral or detrimental at higher concentration, possibly due to toxic effects.

**Table 1.** Etomoxir life span statistical summary.

Experiment	Experiment replicate	Sex	Status	Drug	N	Med	ΔMed (%)	*p*
**E50 conc**	**1**	Male	virgin	-	93	68		
	**1**	Male	virgin	E50	96	72	5.88	0.5177
	**1**	Female	virgin	-	97	62		
	**1**	Female	virgin	E50	98	72	16.1	0.0342
	**1**	Female	mated	-	93	50		
	**1**	Female	mated	E50	96	56	12.0	0.0343
	**2**	Male	virgin	-	85	70		
	**2**	Male	virgin	E50	88	70	0.00	0.6237
	**2**	Female	virgin	-	101	58		
	**2**	Female	virgin	E50	97	60	3.44	0.0344
	**2**	Female	mated	-	98	48		
	**2**	Female	mated	E50	96	59	22.9	5.46E-5
**E100 conc**	**1**	Female	virgin	-	98	63		
	**1**	Female	virgin	E100	97	66	4.76	0.3264
	**1**	Female	mated	-	94	41		
	**1**	Female	mated	E100	94	46	12.2	0.7307
	**2**	Female	virgin	-	95	72		
	**2**	Female	virgin	E100	93	66	−8.33	2.42E-5
	**2**	Female	mated	-	93	48		
	**2**	Female	mated	E100	92	44	−8.33	0.1770

(-) = no drug, EN = NµM Etomoxir. For each type of fly (male virgin, female virgin, female mated), EN is compared to (-). Statistical test is log rank, and the *p* value for significance with two comparisons is 0.025.

In *C. elegans*, gut CPT I regulates feeding behaviour [46]. In mammals, inhibition of FA oxidation by etomoxir (or other FA oxidation inhibitors) reduces hepatic energy status, which is signalled to the brain by the vagus nerve resulting in increased feeding behaviour [47]. This is reminiscent of the increased feeding behaviour often observed in the fly when life span is increased by mifepristone treatment [26,27]. However, no significant effect of 50 µM etomoxir on food intake was observed using EXQ assay (Figure 1c). As discussed above, several lines of evidence point to the midgut as a target for mifepristone’s beneficial effects on life span. In the future, it may be of interest to use conditional RNAi to knock down expression of CPT I specifically in the adult female midgut, to ask if this might increase life span and feeding behaviour, and potentially avoid toxic effects of CPT I inhibition that might occur in other tissues.

### Pioglitazone

Pioglitazone is a well-characterized agonist of mammalian PPARγ [16]. *Drosophila* contains two homologs of mammalian PPARγ, called Eip75B (75B) and Eip78 (E78) [48]. 75B is reported to be required for mating-induced midgut remodelling [14,15]. Zipper et al. found that 7-day treatment of female *Drosophila* with 2 µg/ml pioglitazone dramatically increased the number of ISCs, enteroblasts (EBs) and enterocytes (ECs) in the midgut, in a 75B-dependant manner, suggesting that pioglitazone is a 75B agonist [14]. Jafari et al. reported that treating *Drosophila* with pioglitazone at 10 µg/ml and 20 µg/ml could reduce mortality rates, resulting in an increase in mean longevity in females of 3.8% and males of 2.8% [49].

Here, pioglitazone was tested across a wide range of concentrations for ability to alter life span of virgin and/or mated females, in four experiments (Table 2, experiments A-D). As a positive control, mifepristone was assayed at 200 µg/ml, in virgin and mated females, in two replicate experiments (Figure S2Aa,b; Table 2, experiment A). Consistent with previous results, mifepristone increased life span in virgin females (+31.3% and +30.3%, respectively), and in mated females (+60% and +104.9%, respectively). Pioglitazone was first tested at 1 µg/ml and 2 µg/ml, in virgin females, in two replicate experiments (Figure S2Aa,b; Table 2, experiment A). At 1 µg/ml, pioglitazone caused a small increase in life span (+3.13% and +12.1%, respectively). In contrast, 2 µg/ml pioglitazone increased life span in one replicate (+3.13%), but produced no significant change in the other (−3.03%). Coincident treatment with mifepristone and 1 µg/ml or 2 µg/ml pioglitazone showed no consistent additive or synergistic benefit relative to mifepristone alone (Figure S2Aa,b; Table 2, experiment A). In the next experiment, pioglitazone was tested in virgin females at a range of concentrations from 5 µg/ml to 50 µg/ml, and no significant effect on life span was observed (Figure S2B; Table 2, experiment B). Next, pioglitazone was tested in mated females at lower concentrations (2 µg/ml, 4 µg/ml and 10 µg/ml), in two replicate experiments, and no significant effect on life span was observed (Figure S2Ca,b; Table 2, experiment C). Finally, pioglitazone was tested in virgin and mated females at high concentrations (100 µg/ml and 200 µg/ml), using DMSO as vehicle to enable delivering the higher concentrations, in two replicate experiments (Figure S2Da,b; Table 2, experiment D). However, no consistent effect on life span was observed across the replicate experiments (Table 2, experiment D). Pioglitazone at 200 µg/ml had no significant effect on maximum midgut diameter in virgin females after 12 d treatment (Figure 1d).

**Table 2.** Pioglitazone life span statistical summary.

Experiment	Experiment replicate	Sex	Status	Drug	N	Med	ΔMed (%)	*p*
**A**	**1**	Female	virgin	-	100	64		
**A**	**1**	Female	virgin	M	88	84	31.3	1.56E-20
**A**	**1**	Female	virgin	P1	101	66	3.13	0.00254
**A**	**1**	Female	virgin	P1 + M	103	86	34.4	2.01E-23
**A**	**1**	Female	virgin	P2	95	66	3.13	0.0007
**A**	**1**	Female	virgin	P2 + M	93	86	34.4	7.41E-22
**A**	**1**	Female	mated	-	97	50		
**A**	**1**	Female	mated	M	93	80	60.0	2.77E-19
**A**	**2**	Female	virgin	-	99	66		
**A**	**2**	Female	virgin	M	88	86	30.3	5.46E-21
**A**	**2**	Female	virgin	P1	97	74	12.1	4.23E-6
**A**	**2**	Female	virgin	P1 + M	90	84	27.3	4.59E-29
**A**	**2**	Female	virgin	P2	96	64	−3.03	0.0528
**A**	**2**	Female	virgin	P2 + M	93	82	24.2	1.18E-16
**A**	**2**	Female	mated	-	96	41		
**A**	**2**	Female	mated	M	92	84	104.9	1.84E-22
**B**	**1**	Female	virgin	-	95	62		
**B**	**1**	Female	virgin	P5	93	62	0.00	0.8421
**B**	**1**	Female	virgin	P10	96	58	−6.45	0.9057
**B**	**1**	Female	virgin	P20	100	62	0.00	0.5428
**B**	**1**	Female	virgin	P50	94	66	6.45	0.4264
**C**	**1**	Female	virgin	-	98	63		
**C**	**1**	Female	virgin	M	85	78	23.8	1.86E-9
**C**	**1**	Female	mated	-	94	41		
**C**	**1**	Female	mated	M	94	76	85.4	1.47E-22
**C**	**1**	Female	mated	P2	97	42	2.44	0.6012
**C**	**1**	Female	mated	P4	91	48	17.1	0.7839
**C**	**1**	Female	mated	P10	98	52	26.83	0.2462
**C**	**2**	Female	virgin	-	95	72		
**C**	**2**	Female	virgin	M	91	76	5.56	0.3630
**C**	**2**	Female	mated	-	93	48		
**C**	**2**	Female	mated	M	93	74	54.2	2.36E-15
**C**	**2**	Female	mated	P2	96	50	4.17	0.8450
**C**	**2**	Female	mated	P4	97	48	0.00	0.9275
**C**	**2**	Female	mated	P10	95	54	12.5	0.2543
**D**	**1**	Female	virgin	DMSO	98	68		
**D**	**1**	Female	virgin	P100	93	70	2.94	0.0013
**D**	**1**	Female	virgin	P200	94	74	8.82	4.72E-6
**D**	**1**	Female	mated	DMSO	93	42		
**D**	**1**	Female	mated	P100	100	44	4.76	0.5852
**D**	**1**	Female	mated	P200	96	42	0.00	0.8150
**D**	**2**	Female	virgin	DMSO	98	72		
**D**	**2**	Female	virgin	P100	102	74	2.77	0.1540
**D**	**2**	Female	virgin	P200	94	71	−1.39	0.0048
**D**	**2**	Female	mated	DMSO	94	46		
**D**	**2**	Female	mated	P100	91	40	−13.0	0.6789
**D**	**2**	Female	mated	P200	93	34	−26.1	0.0274

(-) = no drug, M = 200 µg/ml mifepristone/RU486, PN = Nµg/ml pioglitazone. For P1 to P50, the vehicle was ethanol. For P100 and P200, the vehicle was DMSO. The statistical test is log-rank. For experiment A, for each type of fly (virgin or mated), M is compared to (-), PN is compared to (-), PN+M is compared to (-), and the *p* value for significance with 5 comparisons is 0.01. For experiment B, PN is compared to (-), and the *p* value for significance with 4 comparisons is 0.0125. For experiment C, for each type of fly (virgin or mated), M is compared to (-), PN is compared to M, and the *p* value for significance with 4 comparisons is 0.0125. For experiment D, for each type of fly (virgin or mated), PN is compared to DMSO, and the *p* value for significance with two comparisons is 0.025

Taken together, the data suggest no obvious dose-response for pioglitazone effects on life span, and no beneficial effects were observed that were in the range of that typically observed for mifepristone. However, the small magnitude increase in life span observed in virgin females at 1 µg/ml may be worthy of further study, including testing lower concentrations. Joardar et al. examined *Drosophila* larvae with toxic over-expression of TDP-43 in neuronal tissue, and found that 1 µM pioglitazone (~0.36 µg/ml) could rescue larval locomotor dysfunction, and this rescue was dependent on normal expression of 75B and E78 (Joardar et al., 2015). Notably, their study found that pioglitazone was less effective at higher concentrations, consistent with the idea that pioglitazone may have physiological effects that are only observed at low concentrations.

## RH5849

Pioneering studies by Simon et al indicated that ecdysone signalling can negatively regulate adult fly life span [50]. Both male and female flies heterozygous for a null mutation of *EcR* exhibited a 45% increase in mean life span relative to controls, without detectable decrease in fecundity. Moreover, a temperature-sensitive mutation in the gene *mld*, required for normal ecdysone synthesis, increased adult female life span but not adult male life span. The *mld* life span extension could be ‘rescued’ (i.e. reduced) by dietary ecdysone, however, dietary ecdysone had no detectable effect on wild-type female life span in that study [50]. Tricoire et al. used conditional RNAi to knock-down *EcR* expression specifically in adult somatic tissues, and found sex-differences in effects [51]. They reported that mild reduction of *EcR* expression increased life span in males, but decreased life span in females. The negative effect of *EcR* knock-down on female life span was partially rescued by the female-sterile mutation *ovo^D1^*, leading to the suggestion that EcR may be required to suppress a negative effect of the ovary on life span [51]. The differences in results between the Simon et al. and Tricoire et al. studies may be due in part to the fact that the *EcR* mutation will affect ecdysone signalling in all tissues, both during development and in the adult, whereas the conditional RNAi will reduce ecdysone signally specifically in adult somatic tissues. Taken together, the data support an important role for ecdysone signalling in the regulation of adult life span, including interactions with the ovary.

**Figure 2.**
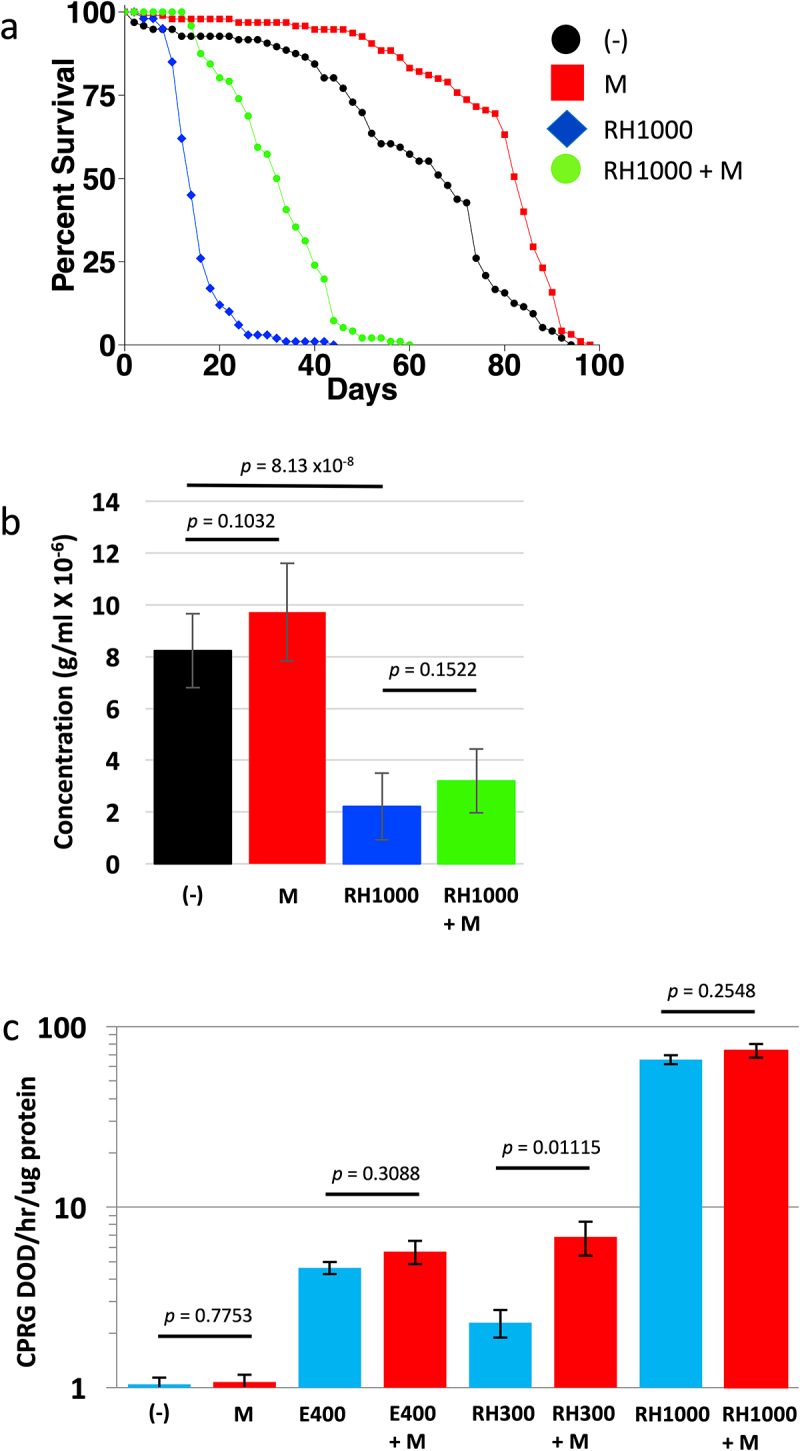
Effect of RH5849 on virgin female life span, food intake, and EcR-responsive reporter. (a) Virgin female life span. Statistical summary for life span presented in Table 3, experiment replicate 1. (b) Virgin female EXQ food intake assay. Bars indicate average and standard deviation of 4 replicates. Statistical test is unpaired, two-sided t-test; *p* value for significance with three comparisons is *p* = 0.0167. (c) Ecdysone receptor (EcR)-responsive LacZ reporter assay. Bars indicate average and standard deviation of 4 replicates. Statistical test is unpaired, two-sided t-test; *p* value for significance with one comparison is *p* = 0.05. (-), no drug. M, 200 µg/ml mifepristone. E400, 400 µg/ml ecdysone. RH300, 300 µg/ml RH5849. RH1000, 1000 µg/ml RH5849.

Ecdysone is reported to regulate the response to mating in female *Drosophila*, including promoting increased egg production and stimulation of cell division in the midgut [14,15,52]. However, whether feeding ecdysone can regulate adult female life span remains unclear. We previously reported that feeding 200 µg/ml ecdysone had no significant effect on virgin female life span [26]. One possible reason for the observed lack of effect is that dietary ecdysone is reported to have poor bioavailability [14,53]. In contrast, the non-steroidal ecdysone mimic RH5849, which is commonly used for pest control, is reported to have good bioavailability, and to activate the ecdysone receptor (EcR) with 30–60 times higher activity than ecdysone [14,54]. Here, virgin females were fed 1000 µg/ml RH5849 throughout adult life span, in two replicate experiments, in the presence and absence of 200 µg/ml mifepristone (Figure 2a; Figure S3; Table 3). Treatment with 200 µg/ml mifepristone alone increased life span, as expected (+23.5% and +26.1%, respectively; Figure 2a; Figure S3; Table 3). Treatment with 1000 µg/ml RH5849 alone dramatically decreased life span (−79.4% and −78.5%, respectively), and co-treatment with 200 µg/ml mifepristone significantly rescued life span (+136% and +157%, respectively; Figure 2a; Figure S3; Table 3). To confirm that the mifepristone rescue of life span was not due to decreased food intake, and therefore decreased intake of RH5849, food intake was assayed using the EXQ assay (Figure 2b). Treatment of flies with 1000 µg/ml RH5849 decreased food intake by ~75%, however mifepristone did not reduce food intake in the presence or absence of RH5849, indicating that the rescue of life span by mifepristone is not due to reduced intake of food or of RH5849 drug. In previous studies, mifepristone has often been observed to significantly increase food intake [24–27], and while a trend towards increased food intake was also observed here, the changes were not statistically significant (Figure 2b). In the future, it may be useful to increase the number of replicates used in the EXQ assay, to further increase sensitivity and specificity.

**Table 3.** RH5849 and mifepristone life span statistical summary.

Experiment replicate	Sex	Status	Drug	N	Med	ΔMed (%)	*p*
**1**	Female	virgin	-	96	68		
**1**	Female	virgin	M	95	84	23.5	2.58E-8
**1**	Female	virgin	RH1000	100	14	−79.4	2.47E-36
**1**	Female	virgin	RH1000 + M	96	33	136	1.44E-25
**2**	Female	virgin	-	96	65		
**2**	Female	virgin	M	88	82	26.1	4.46E-6
**2**	Female	virgin	RH1000	98	14	−78.5	1.26E-42
**2**	Female	virgin	RH1000 + M	102	36	157	2.47E-23

(-) = no drug, M = 200 µg/ml mifepristone/RU486, RH1000 = 1000 µg/ml RH5849. The statistical test is log-rank. For each experiment, M was compared to (-), RH1000 was compared to (-), and RH1000 + M was compared to RH1000. The *p* value for significance with three comparisons is 0.0167.

We previously reported that mifepristone does not compete with dietary ecdysone for activation of an ecdysone receptor (EcR)-responsive transgenic reporter (EcR-LacZ). Here the EcR-LacZ reporter was assayed with RH5849 to ask if it is activated by RH5849 as expected, and whether mifepristone might compete with RH5849. Consistent with our previous results, mifepristone itself did not activate the EcR-LacZ reporter, nor did mifepristone compete with activation of the reporter by 400 µg/ml ecdysone (Figure 2c). RH5849 robustly activated the EcR-LacZ reporter at 300 µg/ml and at 1000 µg/ml, and co-treatment with 200 µg/ml mifepristone did not compete with RH5849 at either concentration (Figure 2c). A significant increase in EcR-LacZ activation was observed when the flies treated with 300 µg/ml RH5894 were co-treated with mifepristone (Figure 2c). Given that mifepristone alone does not activate the reporter (Figure 2c [26];), we conclude this increase is most likely caused by the ability of mifepristone to increase food intake, and thereby increase the intake of RH5849.

Taken together, these results indicate that RH5849 can dramatically reduce adult female life span, and that this life span reduction is partly rescued by mifepristone. This result appears similar to the ability of mifepristone to partly rescue the negative life span effects of the juvenile hormone analog methoprene [26] and HFD [27]. It is important to note that mifepristone does not rescue all toxic challenges that decrease life span. Mifepristone produced little to no rescue of the decreased life span caused by paraquat [27], and mifepristone made the negative life span effects of dietary octopamine more severe [26]. Because RH5849 reduced food intake by ~75%, one possible interpretation is that RH5849 reduces food palatability, in turn causing reduced feeding, undernutrition and shortened life span. In this scenario, the partial rescue of life span by mifepristone might result from its previously observed ability to sometimes increase food intake [24–27]. However, we note that the increase in food intake associated with mifepristone rescue of life span was not statistically significant in these experiments (Figure 2b). In the future, it may be of interest to test lower concentrations of RH5849, to further investigate a possible role for altered food intake.

An alternative possible interpretation for mifepristone rescue of life span in RH5849-treated flies involves interactions with the ecdysone signalling pathway. Because RH5849 is a powerful ecdysone mimic that strongly activates the EcR, one logical hypothesis is that RH5849 reduces adult life span by causing a toxic hyperactivation of the ecdysone signalling pathway. Given that mifepristone is a synthetic steroid that antagonizes both the progesterone receptor and the glucocorticoid receptor in mammals [1,2], it was conceivable that mifepristone might similarly compete with RH5849 for binding to Drosophila EcR. However, because mifepristone did not compete with ecdysone [26] or RH5849 for activation of an EcR-responsive reporter (Figure 2c), it suggests the relevant target for mifepristone is not EcR. One alternative candidate for the target of mifepristone is 75B. Zipper et al. reported that feeding virgin females with 50 µg/ml RH5849 strongly induced ISC mitosis, resulting in dramatic production of new progeny cells, and that this proliferation was dependent upon 75B function in gut progenitor cells [14]. Moreover, that study used the GAL4/GAL80ts system, and the ‘esg’ driver to alter 75B expression in progenitor cells in the midgut. They reported that both 75B over-expression and 75B knock-down by RNAi caused reduced numbers of new ECs and a smaller midgut. Therefore, either activation or inhibition of 75B, such as by direct binding of mifepristone, could be hypothesized to rescue life span by preventing the midgut hypertrophy caused by JH and ecdysone signalling pathways. In the future, it will be of interest to inactivate 75B and other candidate mifepristone targets in the adult female, and ask if this might eliminate the response to mifepristone.

### The GAL4/GAL80ts system and 29°C temperature effects

**Figure 3.**
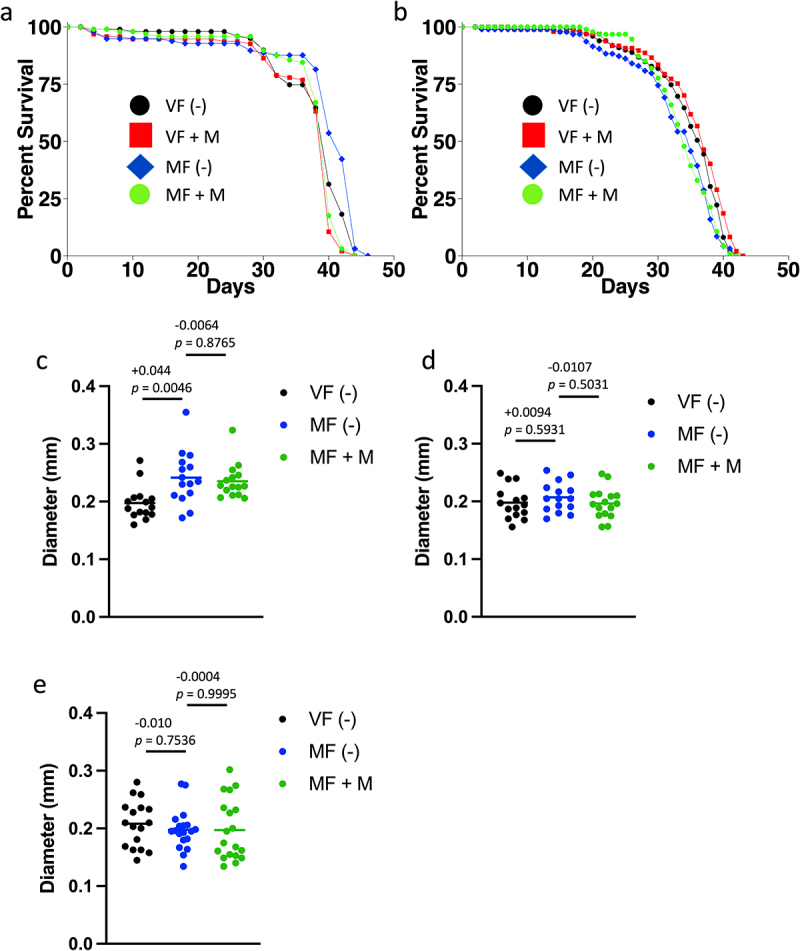
GAL4/GAL80ts system and effects of 29°C temperature on life span and midgut diameter. (a, b) Life span assay of virgin and mated female control groups at 29°C. (a) Genotype *w[1118]; tub-GAL80ts/+; tub-GAL4/+*. (b) Genotype *w[1118]; esg-GAL4/+; tub-GAL80ts/+*. Statistical summary for life span presented in Table 4, experiment replicate 1. (c-e) Maximum midgut diameter assay. (c) Genotype *w[1118]; tub-GAL80ts/+; tub-GAL4/+*. (d) *w[1118]; tub-GAL80ts/UAS-75B-RNAi; tub-GAL4/+*. (e) Genotype *w[1118]; esg-GAL4/+; tub-GAL80ts/+*. Statistical summary for ANOVA and pair-wise comparisons presented in Table S1; the corrected *p* value for significance is *p* = 0.05. The change in mean maximum midgut diameter between samples and the *p* value for significance is presented above the plots. VF, virgin female. MF, mated female. (-), no drug. M, 200 µg/ml mifepristone.

In the GAL4/GAL80ts system [33], tissue specificity is provided by the promoter used to drive GAL4, for example, α*Tubulin*(tub)-GAL4 to yield tissue-general expression, or *esgargot*(esg)-GAL4 to yield expression in midgut progenitor cells. The tub-GAL80ts construct yields tissue-general expression of GAL80ts, which binds to and inactivates GAL4. Upon shift to 29°C, the GAL80ts protein becomes inactive, yielding active GAL4 and conditional expression of transgenes driven by a GAL4 responsive UAS-promoter. In attempt to develop a system for adult-specific inactivation of 75B, a *UAS-75B-RNAi* construct was crossed to the ‘tub’ driver (*tub-GAL4; tub-GAL80ts*), and to the ‘esg’ driver (*esg-GAL4; tub-GAL80ts*). Control flies were generated by crossing the drivers to the *w[1118]* strain. In these pilot experiments, the *UAS-75B-RNAi* line was not yet backcrossed to the *w[1118]* strain, and we note that such backcrossing will be important for the ultimate experiments to make genetic background the same between the control and RNAi groups. Female flies were collected as virgins over 24 hours, shifted to 29°C, and then mated or maintained as virgins, and life span was assayed in the presence and absence of mifepristone, in two replicate experiments for each driver (Figure 3a,b; Figure S4; Table 4). As expected, overall life spans were significantly reduced at 29°C. For example, the median life span of the virgin female control flies maintained at 29°C ranged from 37–40 d (Table 4), whereas median life span of virgin control flies at 25°C in the experiments presented above ranged from 58 to 74 d (Tables 1–3). Surprisingly, the negative effect of mating on life span was reduced or absent (non-significant, ns) in the control flies for both the ‘tub’ driver controls (+5% and −5.25%ns, respectively; Figure 3a; Figure S4c; Table 4), and the ‘esg’ driver controls (−5.45%ns and −6.49%ns, respectively; Figure 3b; Figure S4f; Table 4). Moreover, no beneficial effect of mifepristone was observed in the control flies for either driver, in either of the replicate experiments (Figure 3a,b; Figure S4c,f; Table 4).

**Table 4.** GAL4/GAL80ts life span statistical summary.

Experiment	Experiment replicate	Genotype	Sex	Status	Drug	N	Med	ΔMed (%)	*p*
***GAL80ts;Tub-GAL4 (‘tub’)***									
	**1**	*tub X Eip75B RNAi*	Female	virgin	-	100	32		
	**1**	*tub X Eip75B RNAi*	Female	virgin	M	95	28	−12.5	0.0176
	**1**	*tub X Eip75B RNAi*	Female	mated	-	91	34	6.25	0.6133
	**1**	*tub X Eip75B RNAi*	Female	mated	M	86	28	−17.6	0.0002
	**1**	*tub X w[1118]*	Female	virgin	-	99	40		
	**1**	*tub X w[1118]*	Female	virgin	M	95	40	0.00	0.0403
	**1**	*tub X w[1118]*	Female	mated	-	97	42	5.00	0.0033
	**1**	*tub X w[1118]*	Female	mated	M	97	40	−4.76	2.36E-6
	**2**	*tub X Eip75B RNAi*	Female	virgin	-	102	31		
	**2**	*tub X Eip75B RNAi*	Female	virgin	M	101	26	−16.1	0.0018
	**2**	*tub X Eip75B RNAi*	Female	mated	-	99	34	9.68	0.5244
	**2**	*tub X Eip75B RNAi*	Female	mated	M	100	26	−23.5	2.81E-6
	**2**	*tub X w[1118]*	Female	virgin	-	99	38		
	**2**	*tub X w[1118]*	Female	virgin	M	100	36	−5.26	0.1443
	**2**	*tub X w[1118]*	Female	mated	-	94	36	−5.26	0.0315
	**2**	*tub X w[1118]*	Female	mated	M	96	36	0.00	2.90E-5
***esg-GAL4;GAL80ts (‘esg’)***									
	**1**	*esg X Eip75B RNAi*	Female	virgin	-	96	38		
	**1**	*esg X Eip75B RNAi*	Female	virgin	M	98	38	0.00	0.0721
	**1**	*esg X Eip75B RNAi*	Female	mated	-	97	31	−18.4	2.81E-14
	**1**	*esg X Eip75B RNAi*	Female	mated	M	98	35	12.9	0.0030
	**1**	*esg X w[1118]*	Female	virgin	-	99	37		
	**1**	*esg X w[1118]*	Female	virgin	M	97	37	0.00	0.1022
	**1**	*esg X w[1118]*	Female	mated	-	94	35	−5.45	0.0427
	**1**	*esg X w[1118]*	Female	mated	M	94	34	−2.86	0.9547
	**2**	*esg X Eip75B RNAi*	Female	virgin	-	88	31		
	**2**	*esg X Eip75B RNAi*	Female	virgin	M	94	31	0.00	0.2864
	**2**	*esg X Eip75B RNAi*	Female	mated	-	95	28	−9.68	5.73E-5
	**2**	*esg X Eip75B RNAi*	Female	mated	M	98	29	1.79	0.1228
	**2**	*esg X w[1118]*	Female	virgin	-	64	39		
	**2**	*esg X w[1118]*	Female	virgin	M	50	40	3.90	0.2885
	**2**	*esg X w[1118]*	Female	mated	-	63	36	−6.49	0.0511
	**2**	*esg X w[1118]*	Female	mated	M	54	35	−2.78	0.9341

(-) = no drug, M = 200 µg/ml mifepristone/RU486. The statistical test is log-rank. For each experiment, and for each genotype of fly (‘tub’ X 75B-RNAi, or ‘esg’ X w[1118]), virgin (M) is compared to virgin (-), mated (-) is compared to virgin (-), and mated (M) is compared to mated (-). The *p* value for significance with three comparisons is 0.0167.

The effects of mating and mifepristone on maximum midgut diameter also appeared reduced or absent in the flies maintained at 29°C. For the ‘tub’ driver controls, mating caused a significant increase in average maximum midgut diameter, as expected, however the magnitude of the change (0.044 mm; Figure 3c) was less than that observed in the experiment presented above (0.084 mm; Figure 1d), and no significant effect of mifepristone was detected (Figure 3c). In the experimental group flies, where the ‘tub’ driver activates expression of *UAS-75B-RNAi*, no significant effect of mating on midgut size was detected and no effect of mifepristone was detected (Figure 3d). For the ‘esg’ driver control flies, no significant effect of mating or mifepristone on midgut size was detected (Figure 3e), and therefore analysis of midgut size in ‘esg’ driver experimental flies was not pursued. The more severe inhibition of midgut hypertrophy observed for the ‘esg’ driver control experiment may be because the males used for mating were shifted to 29°C 48 hours prior to mating, whereas for the ‘tub’ driver control experiment, the males were shifted to 29°C coincident with the start of mating (Materials and methods). Taken together, the results with both drivers suggest that the effects of mating and mifepristone on life span and midgut hypertrophy are reduced or absent in flies maintained at 29°C.

**Figure 4.**
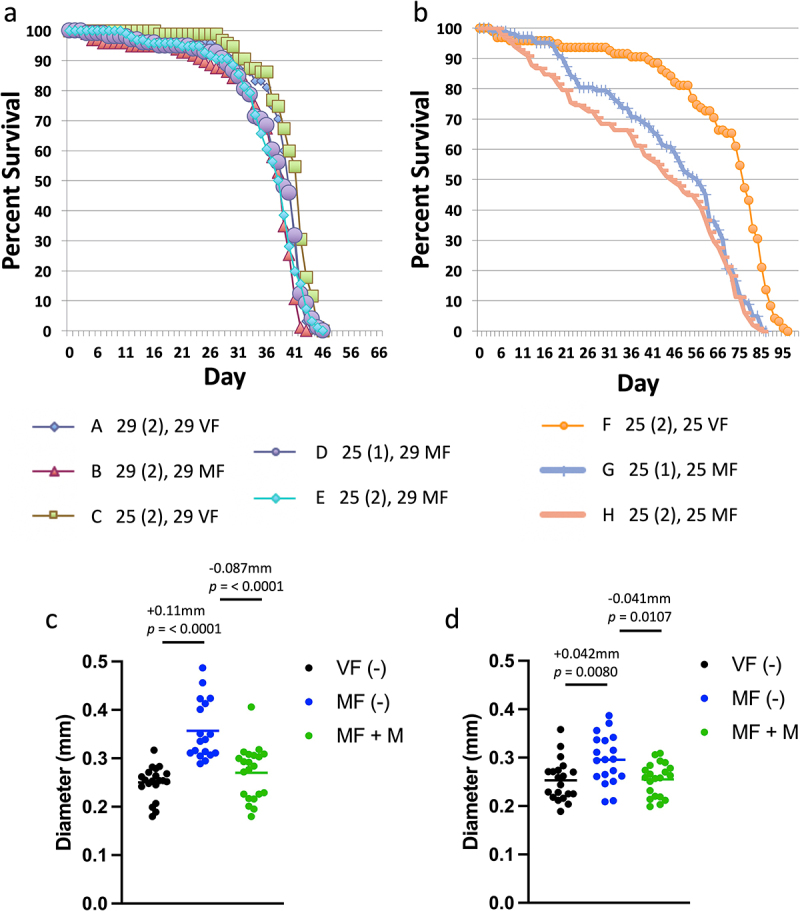
Effect of 29°C temperature on life span and midgut diameter in virgin and mated females. (a, b) Life span assay. The key indicates the 8 groups (a-h), with the temperature of the mating period presented first, followed by the duration of the mating period in days (in parentheses), followed by the temperature used for the remainder of the life span assay. (a) Life span at 29°C. (b) Life span at 25°C. Statistical summary for life span presented in Table 5. (c, d) Maximum midgut diameter assay. Mating was conducted for 2 d at 25°C, and virgins were maintained at 25°C for the same period. Flies were then maintained in presence or absence of mifepristone, as indicated, for 12 d prior to assay. (c) Flies maintained at 25°C after mating period. (d) Flies shifted to 29°C after mating period. Statistical summary for ANOVA and pair-wise comparisons presented in Table S1; the corrected *p* value for significance is *p* = 0.05. The change in mean maximum midgut diameter between samples and the *p* value for significance is presented above the plots. VF, virgin female. MF, mated female. (-), no drug. M, 200 µg/ml mifepristone.

To confirm that the absence of mating and mifepristone effects was not due to the particular genetic background of the GAL4/GAL80ts system strains, the effect of 29°C temperature was assayed in the same *w[1118]* x *y; Elav-GS* hybrid genotype as used in the experiments presented above. Because elevated temperature (≥32°C) has been reported to reduce *Drosophila melanogaster* mating success [55,56], the experiment included flies assayed for life span when the temperature shift occurred either before or after mating was complete (Figure 4; Table 5). In addition, the effect of 1 d versus 2 d mating at 25°C was compared. Comparing flies maintained entirely at 25°C shows the expected difference between virgins (group F) and females mated for 2 d (group H), where median life span is reduced by −40.7% (Figure 4a,b; Table 5). Similarly, comparing flies maintained entirely at 25°C shows a significant difference between virgins (group F) and females mated for only 1 d (group G), where median life span is reduced by −26.7% (Figure 4a,b; Table 5). In contrast, comparing flies maintained entirely at 29°C shows only small magnitude difference between virgins (group A) and females mated for 2 d (group B), where median life span is reduced by −2.56% (Figure 4a,b; Table 5). Notably, the difference between virgin and mated female life span was reduced even when the temperature shift occurred after mating was complete. Comparing virgin females where temperature was shifted from 25°C to 29°C after 2 d (group C), to females mated for 2 d at 25°C and then shifted to 29°C (group E), shows a decrease due to mating of −8.54% (Figure 4a,b; Table 5). Therefore, shifting temperature to 29°C after mating was complete reduced the magnitude of the mating effect on life span from −40.7% to −8.54%, or about 4.8-fold.

**Table 5.** 29°C temperature life span statistical summary.

Group	Sex	Status	Mating period temp (days)	Lifespan temp	N	Med	ΔMed (%)	*p*
**A**	Female	virgin	29 (2)	29	95	39		
**B**	Female	mated	29 (2)	29	95	38	−2.56	0.0006
**C**	Female	virgin	25 (2)	29	95	41		
**D**	Female	mated	25 (1)	29	98	38	−7.32	0.0045
**E**	Female	mated	25 (2)	29	96	38	−8.54	0.0002
**F**	Female	virgin	25 (2)	25	95	75		
**G**	Female	mated	25 (1)	25	102	55	−26.7	3.41E-11
**H**	Female	mated	25 (2)	25	98	46	−40.7	5.55E-15

The statistical test is log-rank. B is compared to A, D is compared to C, E is compared to C, G is compared to F, and H is compared to F. The *p* value for significance with three comparisons is 0.0167.

Shifting the temperature from 25°C to 29°C after mating was complete also reduced the magnitude of mating-induced midgut hypertrophy. As a control, maximum midgut diameter was assayed in virgin females, mated females, and mated females treated with mifepristone, maintained entirely at 25°C (Figure 4b). Consistent with previous results, mating increased maximum midgut diameter by +0.11 mm, and this change was largely inhibited by mifepristone (−0.087 mm; Figure 4c). In parallel, midgut diameter was assayed in virgin females, mated females, and mated females treated with mifepristone, where the flies were shifted to 29°C only after the 2-day mating period was complete (Figure 4d). Under these conditions, mating increased midgut diameter by +0.042 mm, and this change was inhibited by mifepristone (−0.041 mm; Figure 4d). Therefore, shifting temperature to 29°C after mating was complete reduced the magnitude of midgut hypertrophy by approximately one half. It is interesting to note that when comparing group E, where the mated females are shifted to 29°C after mating, to group H, where mated females are maintained at 25°C, there are more early deaths apparent at the 25°C temperature (Figure 4a,b). We speculate these early deaths might result from the greater midgut hypertrophy observed at 25°C. Taken together, these results indicate that sustained exposure to 29°C temperature suppresses mating-induced midgut hypertrophy and reduces or eliminates the difference in life span between virgin and mated females. These effects are similar to the ability of mifepristone to block mating-induced midgut hypertrophy and rescue the negative effect of mating on life span; the major difference being that with 29°C temperature, median life span is greatly reduced, whereas with mifepristone, median life span is greatly increased.

As mentioned above, the GAL4/GAL80ts system has been used with great success to analyse mating-induced cell dynamics and midgut hypertrophy [14,15,20], and consistent with those results, we observe significant midgut hypertrophy at 29°C (Figures 3c, 4d). However, the effects of temperature observed here indicate that the magnitude of hypertrophy is reduced at 29°C relative to 25°C, at least based on the metric of maximum midgut diameter. These results suggest that when designing experiments with GAL4/GAL80ts system it may be optimal to allow mating to occur at 25°C, and to limit the duration of exposure to 29°C. Moreover, mating-induced changes in life span appear to be particularly sensitive to suppression at 29°C. In the future, it may be desirable to use heat-inducible FLP recombinase-based systems, where a several-hour heat pulse causes FLP expression and stable inactivation (or activation) of specific gene function [30,57,58], thereby limiting the duration of exposure of the flies to elevated temperature, especially for assays of life span. Finally, these results may have implications for understanding the potential effects of climate change on dipteran species reproduction and survival.

The present results combined with previous studies also provide some guidance in reducing the confounding life span effects of mifepristone when designing experiments using the Gene-Switch system. Because mifepristone life span effects are largest in mated females, it is helpful to use virgin females and males where possible. Using lower concentrations of mifepristone that are still sufficient to induce the desired gene expression changes is also helpful. The optimal concentration of mifepristone for female life span increase is ~160–200 µg/ml, with much reduced effect observed with concentrations less than ~50 µg/ml [25]; qPCR and/or protein expression analysis should be used to confirm efficient regulation of gene expression at the low dose of mifepristone chosen. It is critical to include controls for mifepristone life span effects using genotypes that lack the target transgene, but contain the Gene-Switch driver and are otherwise as similar to the experimental group as possible. For example, the experimental group can be generated by crossing the driver strain to the target gene strain, and the control group can be generated by crossing the driver strain to the background strain into which the target gene was previously backcrossed. Finally, it is helpful to design experiments where some of the controls involve decreased life span in the presence of mifepristone. For example, if Gene-Switch RNAi of a gene causes increased life span, an ideal control is to ask if Gene-Switch over-expression of the same gene now causes decreased life span, and vice versa.

In summary, the results presented here identify etomoxir as a promising small molecule for further analysis, indicate that mifepristone can partly rescue the negative effect on life span caused by the ecdysone mimic RH5849, and identify 29°C temperature as a suppressor of mating-induced midgut hypertrophy and the life span effects of mating and mifepristone.

## Supplementary Material

Supplemental Material

## Data Availability

The data that support the findings of this study are contained within the paper. Raw survival tables, midgut measurements and spectrophotometer readings are available from the corresponding author upon request.
