# Supplementary material for: A screen of small molecule and genetic modulators of life span in female Drosophila identifies etomoxir, RH5849 and unanticipated temperature effects
Source: Fly (Austin). 2022 Nov 22;16(1):397–413. doi: 10.1080/19336934.2022.2149209 (PMC9683069; doi:10.1080/19336934.2022.2149209)
Supplement: Supplemental Material [file KFLY_A_2149209_SM0792.pdf]

A screen of small molecule and genetic modulators of life span in female Drosophila identifies etomoxir, RH5849 and unanticipated temperature effects

Gary N. Landis, Sebastian Ko, Oscar Peng, Brett Bognar, Michael Khmelkov, Hans S. Bell, John Tower

Supplemental materials

Supplemental figures

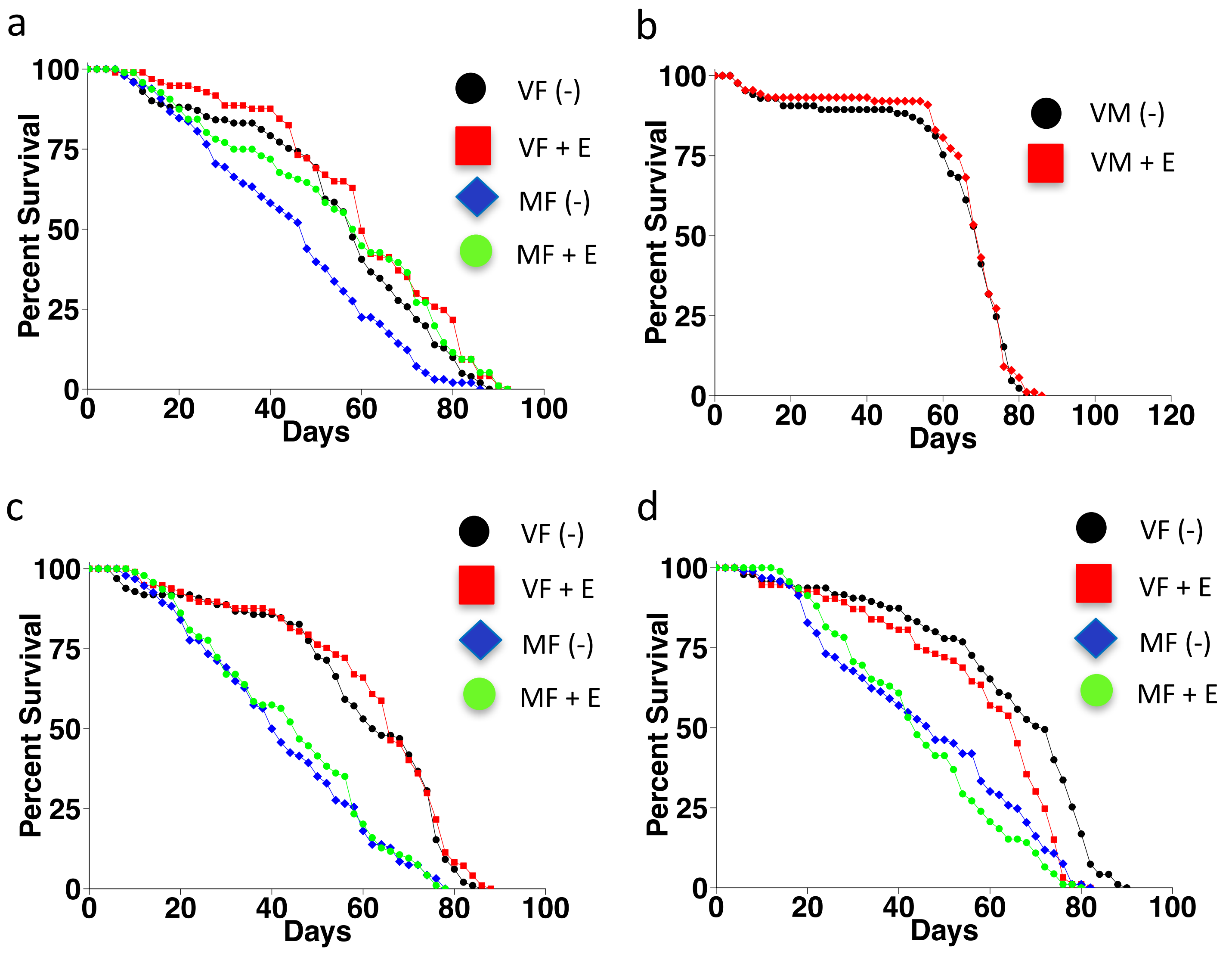

Figure S1. Effect of 50µM etomoxir and 100µM etomoxir on life span. (a) Female life span with 50 Etomoxir (Table 1 E50 Experiment replicate 2). (b) Male life span with 50µM etomoxir (Table 1 E50 Experiment replicate 2). (c) Female life span with 100µM Etomoxir (Table 1 E100 Experiment replicate 1). (d) Female life span with 100µM Etomoxir (Table 1 E100 Experiment replicate 2).

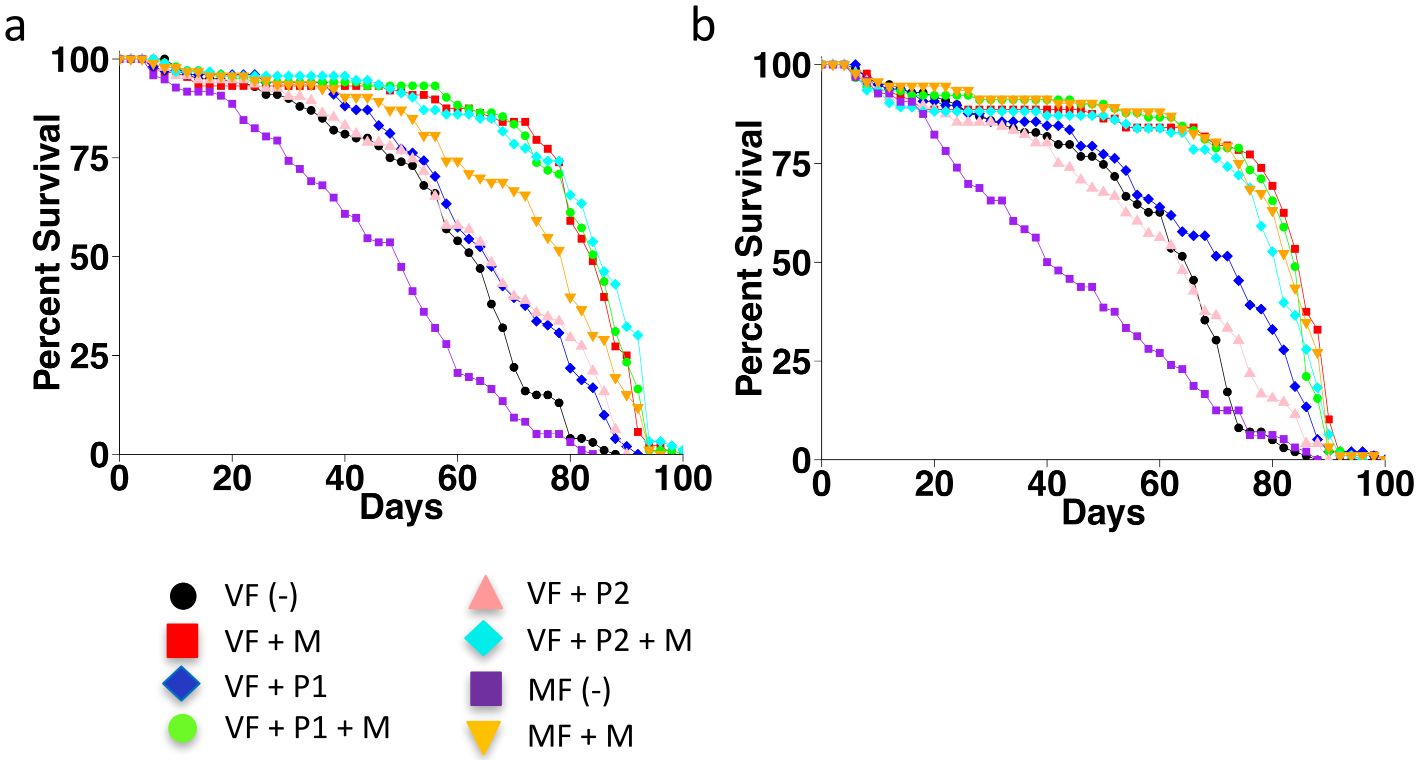

Figure S2A. Effect of pioglitazone on female life span, experiment A. (a) Experiment replicate 1 (Table 2 A-1). (b) Experiment replicate 2 (Table 2 A-2). VF, virgin female. MF, mated female. P1, 1µg/ml pioglitazone. P2, 2µg/ml pioglitazone. M, 200µg/ml mifepristone.

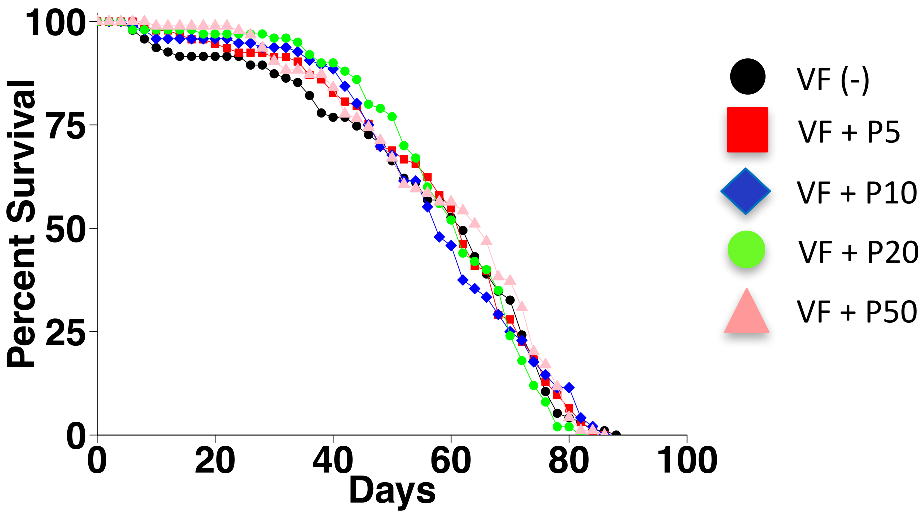

Figure S2B. Effect of pioglitazone on female life span, experiment B (Table 2 B). VF, virgin female. (-), no drug. P5, 5µg/ml pioglitazone. P10, 10µg/ml pioglitazone. P20, 20µg/ml pioglitazone. P50, 50µg/ml pioglitazone.

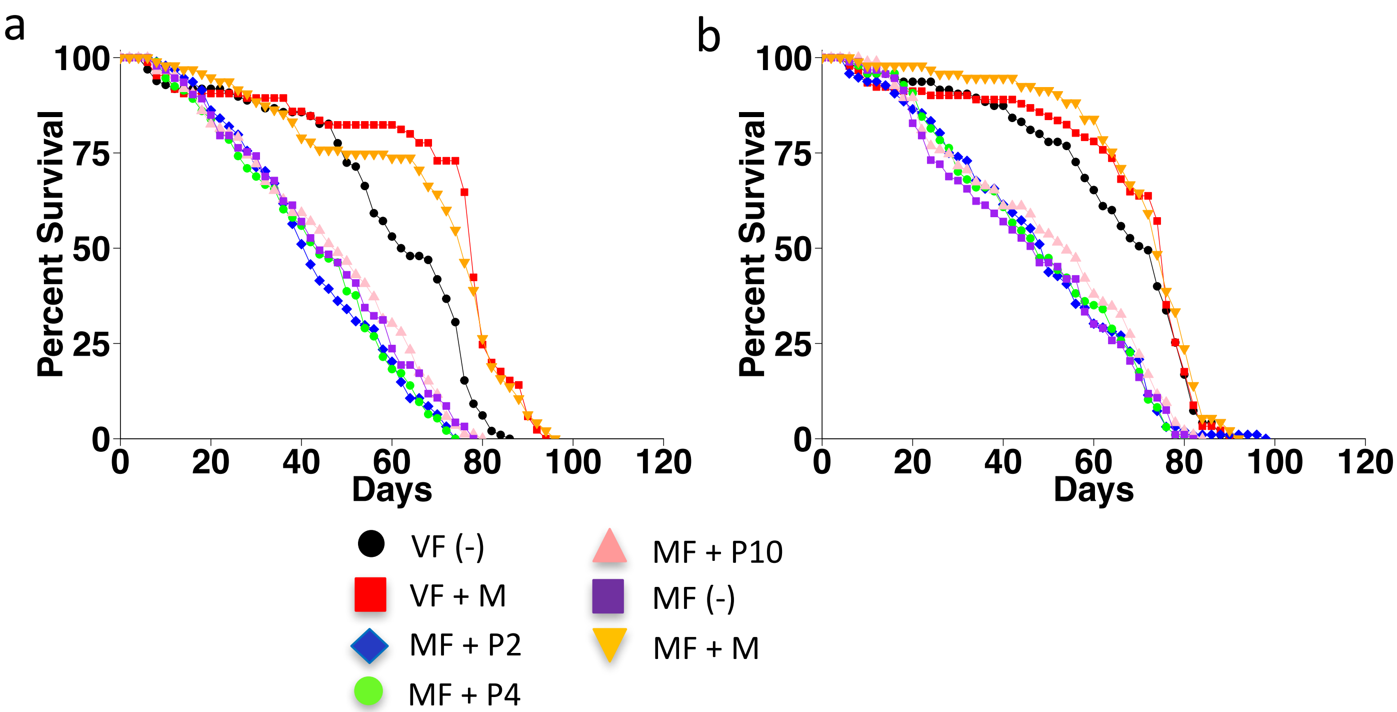

Figure S2C. Effect of pioglitazone on female life span, experiment C (Table 2 C).
(a) Experiment replicate 1 (Table 2 C-1). (b) Experiment replicate 2 (Table 2 C-2). VF, virgin female. MF, mated female. (-) no drug. P2, 2µg/ml pioglitazone. P4, 4µg/ml pioglitazone. P10, 10µg/ml pioglitazone. M, 200µg/ml mifepristone.

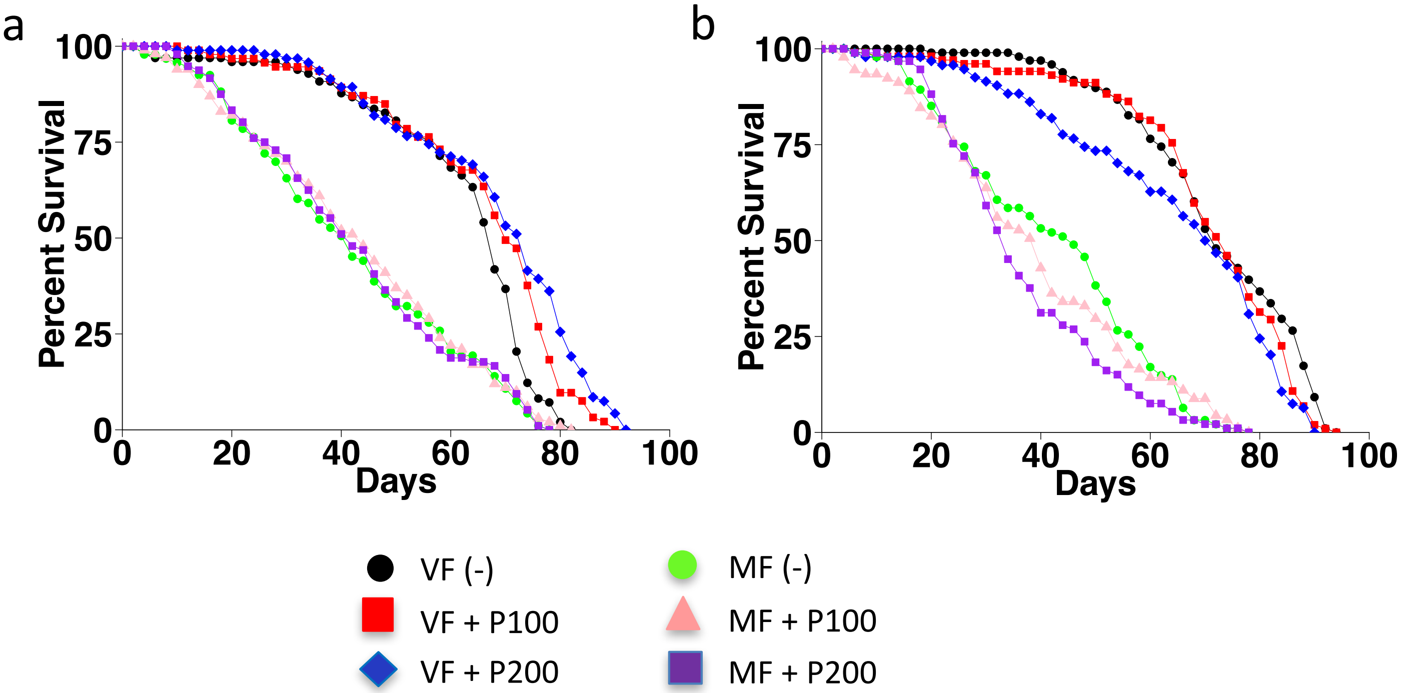

Figure S2D. Effect of pioglitazone on female life span, experiment D (Table 2 D).
(a) Experiment replicate 1 (Table 2 D-1). (b) Experiment replicate 2 (Table 2 D-2). VF, virgin female. MF, mated female. (-) no drug, DMSO vehicle. P100, 100µg/ml Pioglitazone. P200, 200µg/ml Pioglitazone.

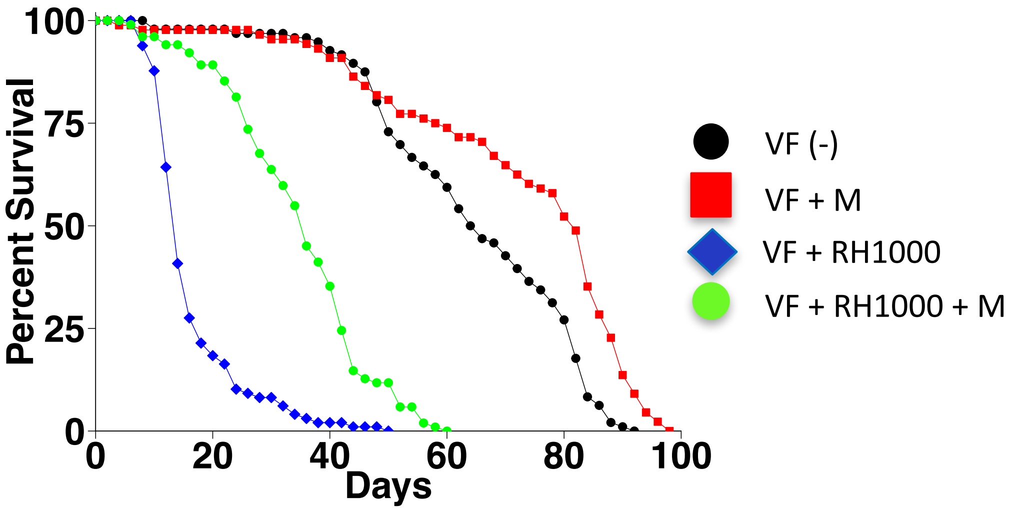

Figure S3. Effect of RH5849 on virgin female life span, experiment replicate 2. VF, virgin female. (-), no drug. M, 200µg/ml mifepristone. RH1000, 1000µg/ml RH5849.

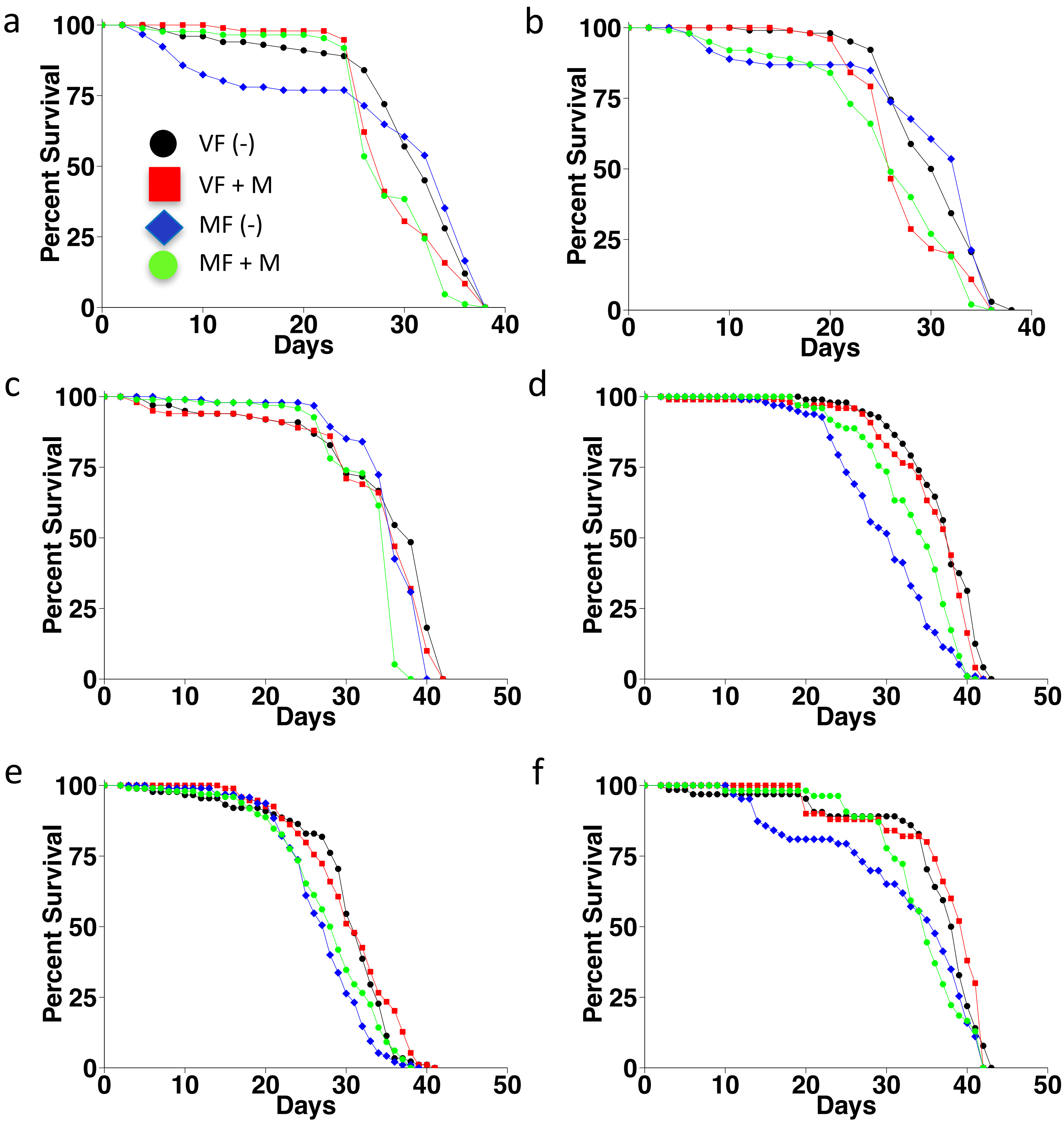

Figure S4. GAL4/GAL80ts system and effects of 29^{0}C temperature on life span. (a) Genotype *w[1118]; tub-GAL80ts/UAS-75B-RNAi; tub-GAL4/+*. Table 4, experiment replicate 1. (b) Genotype *w[1118]; tub-GAL80ts/UAS-75B-RNAi; tub-GAL4/+*. Table 4, experiment replicate 2. (c) Genotype *w[1118]; tub-GAL80ts/+; tub-GAL4/+*. Table 4, experiment replicate 2. (d) Genotype *w[1118]; esg-GAL4/UAS-75B-RNAi; tub-GAL80ts/+*. Table 4, experiment replicate 1. (e) Genotype *w[1118]; esg-GAL4/UAS-75B-RNAi; tub-GAL80ts/+*. Table 4, experiment replicate 2. (f) Genotype *w[1118]; esg-GAL4/+; tub-GAL80ts/+*. Table 4, experiment replicate 2. VF, virgin female. MF, mated female. (-), no drug. M, 200µg/ml mifepristone.

Table S1. Anova Tables

"Table Analyzed" "P midgut diameter assay"
"Data sets analyzed" A-C

"ANOVA summary"
" F" 14.35
" P value" <0.0001
" P value summary" ****
" Significant diff. among means (P < 0.05)?" Yes
" R squared" 0.3602

"Brown-Forsythe test"
" F (DFn, DFd)" "1.712 (2, 51)"
" P value" 0.1907
" P value summary" ns
" Are SDs significantly different (P < 0.05)?" No

"Bartlett's test"
" Bartlett's statistic (corrected)" 1.671
" P value" 0.4336
" P value summary" ns
" Are SDs significantly different (P < 0.05)?" No

"ANOVA table" SS DF MS "F (DFn, DFd)" "P value"
" Treatment (between columns)" 0.06121 2 0.03060 "F (2, 51) = 14.35" P<0.0001
" Residual (within columns)" 0.1087 51 0.002132
" Total" 0.1699 53

"Data summary"
" Number of treatments (columns)" 3
" Number of values (total)" 54

"Number of families" 1
"Number of comparisons per family" 3
Alpha 0.05

"Tukey's multiple comparisons test" "Mean Diff." "95.00% CI of diff." "Below threshold?" Summary "Adjusted P Value"
" VF vs. MF" -0.08360 "-0.1221 to -0.04510" Yes **** <0.0001 A-B

" VF vs. MF + P"	-0.02245	"-0.05816 to 0.01326"	No	ns	0.2913 A-C

" MF vs. MF + P"	0.06115	"0.02308 to 0.09922" Yes	***	0.0009 B-C

"Test details"	"Mean 1"	"Mean 2"	"Mean Diff."	"SE of diff."	n1	n2	q
DF
" VF vs. MF"	0.2180 0.3016 -0.08360	0.01595	19	15	7.413	51
" VF vs. MF + P"	0.2180 0.2405 -0.02245	0.01479	19	20	2.146	51
" MF vs. MF + P"	0.3016 0.2405 0.06115	0.01577	15	20	5.483	51

**"Table Analyzed"	"tub x w[1118] midgut diameter"**
"Data sets analyzed"	A-C

"ANOVA summary"
" F"	6.622
" P value"	0.0032
" P value summary"	**
" Significant diff. among means (P < 0.05)?" Yes
" R squared"	0.2397

"Brown-Forsythe test"
" F (DFn, DFd)"	"1.391 (2, 42)"
" P value"	0.2601
" P value summary"	ns
" Are SDs significantly different (P < 0.05)?" No

"Bartlett's test"
" Bartlett's statistic (corrected)"	3.647
" P value"	0.1615
" P value summary"	ns
" Are SDs significantly different (P < 0.05)?" No

"ANOVA table"	SS	DF	MS	"F (DFn, DFd)" "P value"
" Treatment (between columns)"	0.01695	2	0.008477	"F (2, 42) = 6.622"
P=0.0032
" Residual (within columns)" 0.05376	42	0.001280
" Total"	0.07072	44

"Data summary"
" Number of treatments (columns)" 3
" Number of values (total)"	45

"Number of families"	1

"Number of comparisons per family" 3
Alpha 0.05

"Tukey's multiple comparisons test" "Mean Diff." "95.00% CI of diff." "Below threshold?" Summary "Adjusted P Value"
" VF (-) vs. MF (-)" -0.04400 "-0.07574 to -0.01226" Yes ** 0.0046 A-B

" VF (-) vs. MF + M" -0.03760 "-0.06934 to -0.005861" Yes * 0.0169 A-C

" MF (-) vs. MF + M" 0.006400 "-0.02534 to 0.03814" No ns 0.8765 B-C

"Test details" "Mean 1" "Mean 2" "Mean Diff." "SE of diff." n1 n2 q DF
" VF (-) vs. MF (-)" 0.1977 0.2417 -0.04400 0.01306 15 15 4.763 42
" VF (-) vs. MF + M" 0.1977 0.2353 -0.03760 0.01306 15 15 4.070 42
" MF (-) vs. MF + M" 0.2417 0.2353 0.006400 0.01306 15 15 0.6928 42

"Table Analyzed" "tub x UAS-75B-RNAi midgut diameter"
"Data sets analyzed" A-C

"ANOVA summary"
" F" 0.7470
" P value" 0.4799
" P value summary" ns
" Significant diff. among means (P < 0.05)?" No
" R squared" 0.03358

"Brown-Forsythe test"
" F (DFn, DFd)" "0.03801 (2, 43)"
" P value" 0.9627
" P value summary" ns
" Are SDs significantly different (P < 0.05)?" No

"Bartlett's test"
" Bartlett's statistic (corrected)" 0.09846
" P value" 0.9520
" P value summary" ns
" Are SDs significantly different (P < 0.05)?" No

"ANOVA table" SS DF MS "F (DFn, DFd)" "P value"
" Treatment (between columns)" 0.001045 2 0.0005223 "F (2, 43) = 0.7470" P=0.4799

" Residual (within columns)" 0.03007 43 0.0006993
" Total" 0.03111 45

"Data summary"
" Number of treatments (columns)" 3
" Number of values (total)" 46

"Number of families" 1
"Number of comparisons per family" 3
Alpha 0.05

"Tukey's multiple comparisons test"	"Mean Diff."	"95.00% CI of diff."	"Below threshold?"	Summary	"Adjusted P Value"	
" VF (-) vs. MF (-)"	-0.009467	"-0.03291 to 0.01397"	No	ns	0.5931	A-B
" VF (-) vs. MF + M"	0.001242	"-0.02183 to 0.02431"	No	ns	0.9906	A-C
" MF (-) vs. MF + M"	0.01071	"-0.01236 to 0.03378"	No	ns	0.5031	B-C

"Test details"	"Mean 1"	"Mean 2"	"Mean Diff."	"SE of diff."	n1	n2	q	DF
" VF (-) vs. MF (-)"	0.1979	0.2073	-0.009467	0.009656	15	15	1.386	43
" VF (-) vs. MF + M"	0.1979	0.1966	0.001242	0.009504	15	16	0.1848	43
" MF (-) vs. MF + M"	0.2073	0.1966	0.01071	0.009504	15	16	1.593	43

"Table Analyzed" "esg x w[1118] midgut diameter"
"Data sets analyzed" A-C

"ANOVA summary"
" F" 0.3522
" P value" 0.7048
" P value summary" ns
" Significant diff. among means (P < 0.05)?" No
" R squared" 0.01337

"Brown-Forsythe test"
" F (DFn, DFd)" "2.437 (2, 52)"
" P value" 0.0973
" P value summary" ns
" Are SDs significantly different (P < 0.05)?" No

"Bartlett's test"

" Bartlett's statistic (corrected)"	3.049
" P value"	0.2178
" P value summary"	ns
" Are SDs significantly different (P < 0.05)?"	No

"ANOVA table"	SS	DF	MS	"F (DFn, DFd)"	"P value"
" Treatment (between columns)"	0.001331	2	0.0006654	"F (2, 52) = 0.3522"	P=0.7048
" Residual (within columns)"	0.09823	52	0.001889		
" Total"	0.09956	54			

"Data summary"	
" Number of treatments (columns)"	3
" Number of values (total)"	55

"Number of families"	1
"Number of comparisons per family"	3
Alpha	0.05

"Tukey's multiple comparisons test"	"Mean Diff."	"95.00% CI of diff."	"Below threshold?"	Summary	"Adjusted P Value"	
" VF (-) vs. MF (-)"	0.01043	"-0.02458 to 0.04543"	No	ns	0.7536	A-B
" VF (-) vs. MF + M"	0.01085	"-0.02416 to 0.04586"	No	ns	0.7364	A-C
" MF (-) vs. MF + M"	0.0004211	"-0.03360 to 0.03444"	No	ns	0.9995	B-C

"Test details"	"Mean 1"	"Mean 2"	"Mean Diff."	"SE of diff."	n1	n2	q	DF
" VF (-) vs. MF (-)"	0.2081	0.1976	0.01043	0.01451	17	19	1.016	52
" VF (-) vs. MF + M"	0.2081	0.1972	0.01085	0.01451	17	19	1.057	52
" MF (-) vs. MF + M"	0.1976	0.1972	0.0004211	0.01410	19	19	0.04223	52
